# Cervical Cancer: Associations between Metabolic Parameters and Whole Lesion Histogram Analysis Derived from Simultaneous ^18^F-FDG-PET/MRI

**DOI:** 10.1155/2018/5063285

**Published:** 2018-07-30

**Authors:** Hans-Jonas Meyer, Sandra Purz, Osama Sabri, Alexey Surov

**Affiliations:** ^1^Department of Diagnostic and Interventional Radiology, University of Leipzig, Leipzig, Germany; ^2^Department of Nuclear Medicine, University of Leipzig, Leipzig, Germany

## Abstract

Multimodal imaging has been increasingly used in oncology, especially in cervical cancer. By using a simultaneous positron emission (PET) and magnetic resonance imaging (MRI, PET/MRI) approach, PET and MRI can be obtained at the same time which minimizes motion artefacts and allows an exact imaging fusion, which is especially important in anatomically complex regions like the pelvis. The associations between functional parameters from MRI and ^18^F-FDG-PET reflecting different tumor aspects are complex with inconclusive results in cervical cancer. The present study correlates histogram analysis and ^18^F-FDG-PET parameters derived from simultaneous FDG-PET/MRI in cervical cancer. Overall, 18 female patients (age range: 32–79 years) with histopathologically confirmed squamous cell cervical carcinoma were retrospectively enrolled. All 18 patients underwent a whole-body simultaneous ^18^F-FDG-PET/MRI, including diffusion-weighted imaging (DWI) using *b*-values 0 and 1000 s/mm^2^. Apparent diffusion coefficient (ADC) histogram parameters included several percentiles, mean, min, max, mode, median, skewness, kurtosis, and entropy. Furthermore, mean and maximum standardized uptake values (SUV_mean_ and SUV_max_), metabolic tumor volume (MTV), and total lesion glycolysis (TLG) were estimated. No statistically significant correlations were observed between SUV_max_ or SUV_mean_ and ADC histogram parameters. TLG correlated inversely with p25 (*r*=−0.486, *P*=0.041), p75 (*r*=−0.490, *P*=0.039), p90 (*r*=−0.513, *P*=0.029), ADC median (*r*=−0.497, *P*=0.036), and ADC mode (*r*=−0.546, *P*=0.019). MTV also showed significant correlations with several ADC parameters: mean (*r*=−0.546, *P*=0.019), p10 (*r*=−0.473, *P*=0.047), p25 (*r*=−0.569, *P*=0.014), p75 (*r*=−0.576, *P*=0.012), p90 (*r*=−0.585, *P*=0.011), ADC median (*r*=−0.577, *P*=0.012), and ADC mode (*r*=−0.597, *P*=0.009). ADC histogram analysis and volume-based metabolic 18F-FDG-PET parameters are related to each other in cervical cancer.

## 1. Introduction

Cervical cancer is the third most commonly diagnosed cancer and the fourth leading cause of cancer death in females worldwide [1].

Magnetic resonance imaging (MRI) has been established as the best imaging modality for staging of cervical cancers due to its excellent soft tissue contrast [2]. Furthermore, MRI can provide information regarding tumor microstructure by diffusion-weighted imaging (DWI). The principle hypothesis is that DWI can quantify the free movement of protons (Brownian molecular movement) by using apparent diffusion coefficients (ADC) [3]. This movement is hindered predominantly by cell membranes. In fact, previous studies showed that ADC inversely correlated with cell count in several malignant and benign lesions [4].

Another clinically important functional imaging modality is ^18^F-fluorodeoxyglucose positron emission tomography (FDG-PET), which reflects tumor glucose-metabolism [5]. The FDG-uptake in tumor tissue is associated with the increased expression of glucose transporters (GLUT), mainly subtype GLUT-1 [6]. Clinically, ^18^F-FDG-uptake is semiquantified by standardized uptake values (SUV). Moreover, it has been shown that volume-based metabolic PET parameters, such as metabolic tumor volume (MTV) and total lesion glycolysis (TLG), might provide additional information regarding tumor behavior [7]. MTV and TLG have been reported as possible prognostic factors, for example, for lung cancer or laryngeal carcinoma. In cervical cancer, for example, MTV was the only parameter to be of prognostic relevance in a multivariate analysis performed by Hong et al. [8].

Presumably, functional parameter derived from PET and from MRI, albeit reflecting slightly different tumor aspects, might be linked to each other [9]. As a hypothesis, a cell-rich tumor might also express more GLUT-transporters within their cell membranes, and hence, an association between ADC and SUV values might exist.

In fact, this was studied by various investigations in several different tumor entities like esophageal or breast cancer [9–13]. However, in a recent meta-analysis, comprising 35 studies, only a weak inverse correlation coefficient of *r*=−0.30 was identified over all various investigated tumors [9].

Regarding cervical cancer, there are inconclusive results [10, 14–16].  Table 1 summarizes the published data about reported correlations between ADC and SUV values. So, Brandmaier et al. identified an inverse correlation between SUV_max_ and ADC_min_ (*r*=−0.532, *P*=0.05) [10], whereas most authors did not [14–16].

An emergent imaging analysis, namely, ADC histogram analysis, which is based on pixel distribution, is used to improve tumor heterogeneity in DWI-MRI assessment. Every voxel of a region of interest is issued into a histogram and thusly statistically information about the tumor is provided. Typically parameters are percentiles, median, mode, skewness, kurtosis, and entropy [17]. It is acknowledged that heterogeneity displayed by the histogram might be reflected by tumor microstructure heterogeneity, and therefore, a better reflection of tumor biology may be possible [17]. The histogram analysis approach has been applied in other tumors, for example, in prostate cancer. For example, Liu et al. characterized histogram variables of ADC as predictors for the aggressiveness of prostate cancer [18]. In a study of Shindo et al., ADC histogram analysis has been described as helpful in differentiating pancreatic adenocarcinomas from neuroendocrine tumors [19]. Regarding cervical cancer, there are only few reports compared metabolic parameters of ^18^F-FDG-PET and ADC histogram analysis. For instance, Ueno et al. evaluated the prognostic value of SUV, MTV and TLG, and ADC histogram analysis for tumor response to therapy and event-free survival in patients with cervical cancer [20]. It has been shown that pretreatment volume-based metabolic ^18^F-FDG-PET parameters may have better potential than ADC histogram analysis for predicting treatment response and survival in these patients [20]. The main drawback of this study was that data from PET and MRI were obtained sequentially and not simultaneously; thus, the results of this study may have been influenced by this fact.

The aim of our study was to elucidate possible associations between ADC histogram-based parameters and ^18^F-FDG-PET parameters derived from simultaneous PET/MRI in cervical cancer.

## 2. Materials and Methods

This prospective study was approved by the local research ethics committee.

### 2.1. Patients

Overall, 18 female patients (age range: 32–79 years; mean age: 55.4 years) with histopathologically confirmed squamous cell cervical carcinoma were enrolled. Inclusion criteria were a staging investigating with a body simultaneous ^18^F-FDG-PET/MRI before any form of treatment.

Table 2 gives an overview about the patients and the different clinical pathological stages.

### 2.2. PET/MRI

All 18 patients underwent a whole-body simultaneous ^18^F-FDG-PET/MRI (Biograph mMR-Biograph, Siemens Healthcare Sector, Erlangen, Germany) which was performed from the upper thigh to the skull for 4 minutes per bed position. PET images were reconstructed using the iterative ordered subset expectation maximization algorithm with 3 iterations and 21 subsets, a Gaussian filter with 4 mm full width at half maximum (FWHM), and a 256 × 256 image matrix. Attenuation correction of the PET data was performed using a four-tissue (fat, soft tissue, air, and background) model attenuation map, which was generated from a Dixon-Vibe MR sequence according to previous description.

Radiotracer administration was performed intravenously after a fasting period of at least 6 hours with a body weight-adapted dose of ^18^F-FDG (4 MBq/kg; range: 152–442 MBq; mean ± std: 285 ± 70 MBq). PET/MRI image acquisition started on average 122 minutes after ^18^F-FDG application. Due to radiotracer elimination via the urinary tract, which may influence evaluation of pelvic PET images, all patients received a bladder catheter prior to PET/MRI examination.

Image analysis was performed on the dedicated workstation of Hermes Medical Solutions, Sweden. For each tumor, maximum and mean SUV (SUV_max_ and SUV_mean_), total lesion glycolysis (TLG), and metabolic tumor volume (MTV) were determined on PET images. MTV was defined as total tumor volume with an SUV ≥ 2.5 and was calculated automatically. TLG was also calculated automatically by multiplying the MTV of the primary tumor by its SUV_mean_.

**Figure 1:**
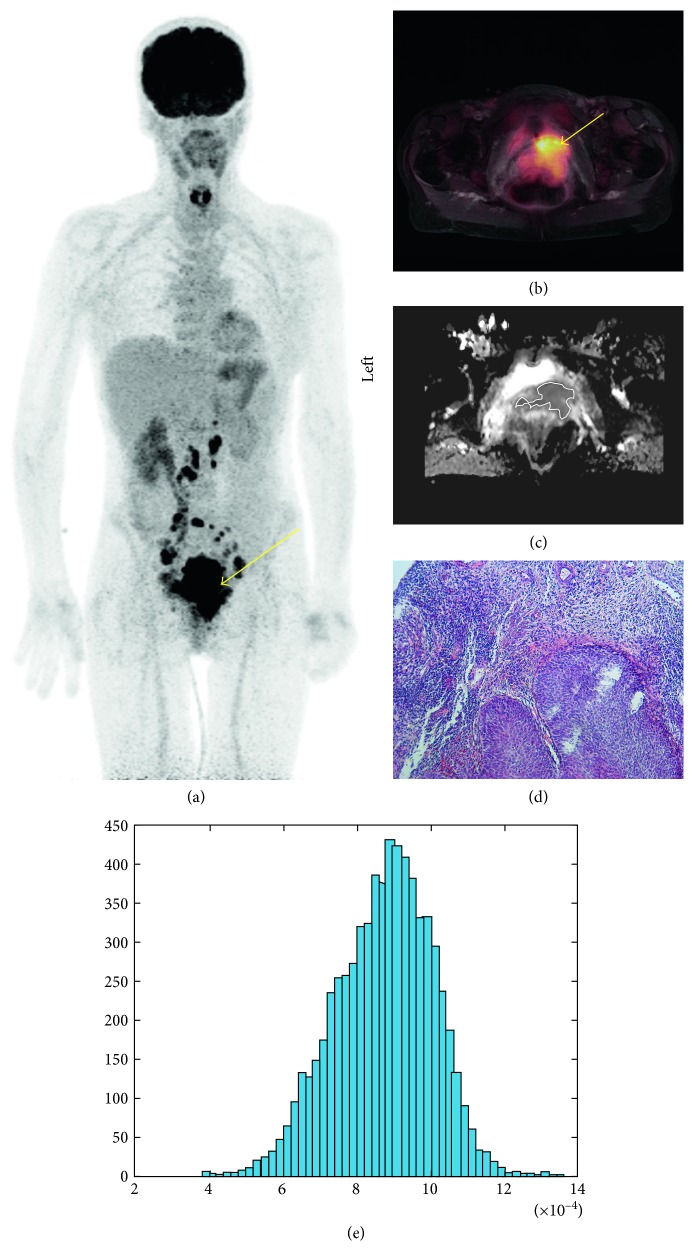
Imaging and histopathological findings in a case of cervical cancer. (a) ^18^F-FDG-PET of a 57-year-old woman with locally advanced cervical cancer (arrow). (b) Fused ^18^F-FDG-PET/MRI image demonstration of the metabolic active uterine cervical cancer (arrow). Calculated ^18^F-FDG-PET parameters are as follows: SUV_max_ = 8.77, SUV_mean_ = 4.66, SUV median = 4.32, TLG = 92.91, and MTV = 19.96. (c) ADC map of the tumor with a ROI. (e) ADC histogram. The histogram analysis parameters (×10^−3^ mm^2^·s^−1^) are as follows: ADC_min_ = 0.36, ADC_mean_ = 0.87, ADC_max_ = 1.36, p10 = 0.7, p25 = 0.78, p75 = 0.96, p90 = 1.03, median = 0.88, and mode = 0.93. Histogram-based characteristics are as follows: kurtosis = 2.96, skewness = −028, and entropy = 4.72. (d) Histopathological examination (hematoxylin and eosin-stained specimen) after tumor biopsy reveals a G2 cervical cancer.

In all cases, pelvic MRI was performed. Our investigation protocol included the following sequences: transverse T2 turbo spin echo (TSE) sequence (TR/TE: 5590/105), sagittal T2 TSE sequence (TR/TE: 4110/131), transverse T1 TSE sequence (TR/TE:1310/12), transverse T1 TSE after intravenous application of contrast medium (0.1 mmol/kg body weight Gadobutrol, Bayer Healthcare, Germany) (TR/TE: 912/12), and sagittal postcontrast T1 TSE (TR/TE: 593/12). Additionally, diffusion-weighted imaging was performed using an echo-planar imaging (EPI) sequence (b0 and b1000 s/mm^2^, TR/TE: 4900/105).  Figure 1 shows an exemplary patient of our patient sample.

### 2.3. Histogram Analysis of ADC Values

Automatically generated ADC maps were transferred in DICOM format and processed offline with custom-made Matlab-based application (The Mathworks, Natick, MA) on a standard windows-operated system. The ADC maps were displayed within a graphical user interface (GUI), which enables the reader to scroll through the slices and draw a volume of interest (VOI) at the tumor's boundary (whole-lesion measure). All measurements were performed by two authors blinded to each other (AS, HJM, 15 and 2 years of radiological experience). The ROIs were modified in the GUI and saved (in Matlab-specific format) for later processing. After setting the ROIs, following parameters were calculated and written in a spreadsheet format: ROI volume (cm^3^), mean (ADC_mean_), maximum (ADC_max_), minimum (ADC_min_), ADC median, 10th (p10 ADC), 25th (p25 ADC), 75th (p75 ADC), 90th (p90 ADC) percentile, and mode (ADC mode). Additionally, histogram-based characteristics of the ROI—kurtosis, skewness, and entropy—were calculated.

### 2.4. Statistical Analysis

Statistical analysis was performed using SPSS 23.0 (SPSS Inc, Chicago, IL). Collected data were evaluated by means of descriptive statistics. The data were not normally distributed according to Kolmogorow–Smirnow test. Therefore, Spearman's correlation coefficient (*p*) was used to analyze associations between investigated parameters. Interreader variability was assessed with intraclass coefficients. *P* values < 0.05 were taken to indicate statistical significance.

## 3. Results

The investigated ADC histogram showed a good interreader variability, ranging from ICC = 0.705 for entropy to ICC = 0.959 for ADC median (Table 3).

Table 4 shows results of correlation analysis between the investigated PET and ADC parameters. No statistically significant correlations were observed between SUV_max_ or SUV_mean_ and ADC histogram parameters.

TLG correlated inversely with p25 (*r*=−0.486, *P*=0.041), p75 (*r*=−0.490, *P*=0.039), p90 (*r*=−0.513, *P*=0.029), ADC median (*r*=−0.497, *P*=0.036), and ADC mode (*r*=−0.546, *P*=0.019). MTV also showed significant correlations with several ADC parameters as follows: mean (*r*=−0.546, *P*=0.019), p10 (*r*=−0.473, *P*=0.047), p25 (*r*=−0.569, *P*=0.014), p75 (*r*=−0.576, *P*=0.012), p90 (*r*=−0.585, *P*=0.011), ADC median (*r*=−0.577, *P*=0.012), and ADC mode (*r*=−0.597, *P*=0.009). Finally, histogram-based parameters—skewness, kurtosis and entropy—did not correlate with PET parameters.

## 4. Discussion

To the best of our knowledge, this is the first study elucidating possible correlations between ADC histogram analysis and complex ^18^F-FDG-PET parameters derived from simultaneous PET/MRI in cervical cancer.

Pretherapeutic tumor staging in cervical cancer is of great importance. MRI is the best imaging modality to estimate regional tumor extent, with identification of tumor infiltration into the adjacent organs/tissues within the female pelvis [2]. Hybrid imaging, in terms of PET/CT, has been shown to be superior to other conventional imaging modalities (MRI, CT) for the identification of nodal or distant metastatic spread [21]. Consequently, the combination of both, namely, a simultaneous PET/MRI, has been described as valuable imaging modality for whole-body tumor staging of cervical cancer patients providing improved treatment planning when compared to MRI alone [22]. Furthermore, our own preliminary data show that simultaneous PET/MRI is a valuable imaging modality to reflect histopathologic parameters like cellularity and proliferation index in cervical cancer [14].

Additionally, functional MRI, as well as ^18^F-FDG-PET can provide information about tumor biology in a different fashion. ADC values derived from DWI are mainly influenced by cellularity, whereas SUV values derived from FDG-PET are mainly influenced by GLUT-1 overexpression within cell membranes and enhanced activity of tumor hexokinase [4, 14, 23].

Presumably, parameters from PET and MRI might be associated with each other due to the fact that a more cell-dense tumor also might express more GLUT-1 or may have an increased enzymatic activity [9]. However, a recent meta-analysis identified only a weak inverse correlation (*r*=−0.30) between SUV and ADC values pooling various tumors in oncologic imaging [9]. Regarding cervical cancer, the studies, which investigated associations between ADC and SUV values, showed inconclusive results [10, 14–16]. Only one study found an inverse correlation between SUV_max_ and ADC_min_ (*r*=−0.532) [10], whereas most authors could not identify linear correlations between these parameters, indicating that they might reflect different tumor aspects [14–16].

The present study identified that several ADC histogram parameters were associated with volume-based metabolic PET parameters, namely, MTV and TLG. In good agreement with the literature, there were no correlations between ADC parameters and SUV values in the current patient sample. Therefore, our results suggest that ADC histogram analysis parameters and TLG and MTV are more sensitive to reflect relationships between ^18^F-FDG-PET and DWI than the widely used SUV and “conventional” ADC values. Furthermore, our study may explain negative results of the previous investigations. Moreover, in the present study, ADC values were obtained as a whole-lesion measurement, whereas in most studies [10, 14–16], only one slice was used for calculation and might therefore not be representative for the whole tumor. According to Kyriazi et al., whole-lesion measurement might be more beneficial than the conventional one slide approach since pixel-by-pixel ADC histograms through the entire tumor volume include different microenvironments of diffusivity, which may be masked by mean ADC analysis [24].

Furthermore, histogram-based analysis has been evaluated to have an excellent interobserver agreement [25, 26]. Additionally, it could clearly discriminate between tissue affected with cancer and physiological cervical tissue [25]. Finally, it could distinguish different FIGO stages: with increasing skewness, kurtosis, and entropy in the advanced stages indicating higher tumor heterogeneity in those lesions [26].

Interestingly, ADC histogram analysis parameters correlated with some histopathological features in cervical cancer. For example, entropy was associated with p53 expression [27]. Moreover, Meng et al. identified that ADC histogram parameters can predict tumor recurrence after radiochemotherapy with an area under the curve 0.85 [28]. In another study, it was identified that skewness and several percentiles derived from ADC maps were significantly different between squamous cell and adenocarcinomas of the uterine cervix and, therefore, ADC histogram analysis might aid in discrimination of the entities [29]. In fact, as reported previously, skewness was significantly higher for squamous cell carcinomas than adenocarcinomas and was higher in poorly differentiated tumors [29].

Regarding ^18^F-FDG-PET, pretreatment SUV_max_ and MTV have been reported to be associated with tumor prognosis [30, 31]. So MTV had a hazard ratio of 3.15 for disease-free survival [31], and SUV_max_ of the primary tumor was the only identified prognostic factor in a multivariate analysis [30]. Furthermore, TLG was also associated with the overall survival in locally advanced cervical cancer [32]. However, it might be of limited use for primary diagnosis in early stage carcinomas since ^18^F-FDG-PET only has little value in the routine pretreatment assessment in patients with early FIGO stages [33]. However, there are promising histopathological methods to better understand underlying microstructure changes, which can be displayed with PET imaging [34].

Overall, our report indicates that for further analyses about associations between DWI and PET and as well between PET, DWI, and histopathology in several tumors, ADC histogram analysis and volume-based metabolic PET parameters like TLG/MTV should be obtained.

There are several limitations of the present study to address. Firstly, it is a retrospective study with possible known bias. However, MRI and ^18^F-FDG-PET were measured by two different readers, blinded to each other. Secondly, the patient sample is relatively small. Thirdly, only squamous cell carcinomas were evaluated.

In conclusion, the present study shows that ADC histogram analysis and volume-based metabolic ^18^F-FDG-PET parameters are related to each other and might, therefore, reflect similar tumor behavior of cervical cancer. The next step would be to assess the value of these simultaneous PET/MRI parameters for predicting treatment response and survival in cervical cancer patients.

## Figures and Tables

**Table 1:** Overview about published literature regarding correlation analysis between DWI and FDG-PET.

Author	Number of patients	Analyzed parameters	Correlation
Ho et al. [15]	33	ADC_min, mean_, SUV_max, mean_	No statistically significant correlations

Sun et al. [16]	35	ADC_min, mean_, SUV_max, mean_	No significant correlation between SUV_max_ and ADC_min_ (*r*=−0.074, *P*=0.501) or between SUV_mean_ and ADC_mean_ (*r*=−0.505, *P*=0.201) across all 35 primary tumors; for the 28 squamous cell carcinomas, there was also no significant correlation between SUV_max_ and ADC_min_ (*r*=−0.363, *P*=0.342) or between SUV_mean_ and ADC_mean_ (*r*=−0.354, *P*=0.150)

Wang et al. [35]	30	ADC_min, mean_, SUV_max, mean_	No statistically significant correlations between ADC and SUV fractions

Brandmaier et al. [10]	31 (14 primary, 17 recurrence)	ADC_min, mean_, SUV_max, mean_	SUV_max_ versus ADC_min_ (*r*=−0.532, *P*=0.05) in primary tumors. Primary metastasis showed weak inverse correlations for SUV_max_ and ADC_min_ (*r*=−0.362, *P*=0.05) and moderate correlations for SUV_mean_ and ADC_min_ (*r*=−0.403, *P*=0.03)

Pinker et al. [36]	11	ADC_mean_, SUV_max_	No significant correlations

Surov et al. [14]	21	ADC_min, mean, max_, SUV_max, mean_	No significant correlations between ADC and SUV fractions

Lai et al. [37]	29	MTV, functional diffusion volume	Significant differences regarding MTV and functional diffusion volume derived from ADC maps

**Table 2:** Clinical data of the investigated patients.

Case	Age	Tumor grade	T stage	N stage	M stage
1	63	G2	2b	1	0
2	76	G3	4	0	0
3	65	G2	2b	0	0
4	63	G3	4	1	1
5	34	G3	2b	1	0
6	57	G2	4	1	1
7	53	G3	2b	0	0
8	32	G2	4	1	0
9	32	G2	2b	0	0
10	54	G2	3a	2	0
11	79	G3	4	1	0
12	52	G1	4	0	0
13	37	G3	2b	1	1
14	72	G3	4	0	0
15	46	G2	2b	1	1
16	71	G2	4	1	1
17	50	G2	2b	1	1
18	61	G2	4	1	0

**Table 3:** Interreader variability with intraclass coefficients of the investigated ADC parameters.

Parameter	ICC
ADC_mean_	0.870
ADC_min_	0.947
ADC_max_	0.920
ADC P10	0.727
ADC P25	0.844
ADC P75	0.804
ADC P90	0.803
ADC median	0.959
ADC mode	0.917
Kurtosis	0.859
Skewness	0.792
Entropy	0.705

ICC, intraclass coefficient.

**Table 4:** Correlation between ADC histogram parameters and ^18^F-FDG-PET parameters in cervical cancer. Spearman's rho correlation coefficient was used.

		SUV_max_	SUV_mean_	SUV_median_	TLG	MTV
Mean ADC	*p* (rho)	−0.134	−0.215	−0.336	−0.461	**−0.546**
	*P*	0.595	0.392	0.173	0.054	**0.019**
Min ADC	*p* (rho)	−0.218	−0.213	−0.282	−0.219	−0.257
	*P*	0.385	0.396	0.257	0.382	0.303
Max ADC	*p* (rho)	−0.044	−0.166	−0.176	0.166	0.162
	*P*	0.861	0.510	0.484	0.510	0.521
P10 ADC	*p* (rho)	−0.183	−0.223	−0.332	−0.413	**−0.473**
	*P*	0.468	0.373	0.179	0.088	**0.047**
P25 ADC	*p* (rho)	−0.150	−0.214	−0.329	**−0.486**	**−0.569**
	*P*	0.553	0.395	0.182	**0.041**	**0.014**
P75 ADC	*p* (rho)	−0.142	−0.244	−0.354	**−0.490**	**−0.576**
	*P*	0.575	0.329	0.150	**0.039**	**0.012**
P90 ADC	*p* (rho)	−0.215	−0.275	−0.361	**−0.513**	**−0.585**
	*P*	0.392	0.270	0.142	**0.029**	**0.011**
Median ADC	*p* (rho)	−0.153	−0.244	−0.368	**−0.497**	**−0.577**
	*P*	0.544	0.329	0.133	**0.036**	**0.012**
Mode ADC	*p* (rho)	−0.225	−0.157	−0.261	**−0.546**	**−0.597**
	*P*	0.370	0.533	0.296	**0.019**	**0.009**
Kurtosis	*p* (rho)	−0.150	−0.148	−0.117	0.288	0.284
	*P*	0.553	0.559	0.645	0.247	0.254
Skewness	*p* (rho)	−0.095	−0.054	−0.004	0.149	0.142
	*P*	0.708	0.832	0.987	0.556	0.573
Entropy	*p* (rho)	0.071	−0.036	−0.049	0.084	0.172
	*P*	0.779	0.887	0.848	0.742	0.494

Significant correlations are highlighted in bold.

## Data Availability

The anonymous patient data used to support the findings of this study are available from the corresponding author upon request.

## Abbreviations

MRI: Magnetic resonance imaging
DWI: Diffusion-weighted imaging
ADC: Apparent diffusion coefficient
FDG-PET: ^18^F-fluorodeoxyglucose positron emission tomography
TLG: Total lesion glycolysis
MTV: Mean tumor volume
GLUT: Glucose transporters
SUV: Standardized uptake value.
